# Angiotensin II produces nociceptive behavior through spinal AT1 receptor-mediated p38 mitogen-activated protein kinase activation in mice

**DOI:** 10.1186/1744-8069-9-38

**Published:** 2013-07-31

**Authors:** Wataru Nemoto, Osamu Nakagawasai, Fukie Yaoita, Syu-Ichi Kanno, Shin Yomogida, Masaaki Ishikawa, Takeshi Tadano, Koichi Tan-No

**Affiliations:** 1 Department of Pharmacology, Tohoku Pharmaceutical University, 4-4-1 Komatsushima, Aoba-ku, Sendai 981-8558, Japan; 2 Department of Clinical Pharmacotherapeutics, Tohoku Pharmaceutical University, Aoba-ku, Sendai 981-8558, Japan; 3 Laboratory of Environmental and Health Sciences, College of Medical Pharmaceutical and Health Sciences, Kanazawa University, Kanazawa 920-1192, Japan

## Abstract

**Background:**

It has been demonstrated that angiotensin II (Ang II) participates in either the inhibition or the facilitation of nociceptive transmission depending on the brain area. Neuronal Ang II is locally synthesized not only in the brain, but also in the spinal cord. Though the spinal cord is an important area for the modulation of nociception, the role of spinal Ang II in nociceptive transmission remains unclear. Therefore, in order to elucidate the role of Ang II in nociceptive transmission in the spinal cord, we examined the effect of intrathecal (i.t.) administration of Ang II into mice.

**Results:**

I.t. administration of Ang II produced a behavioral response in mice mainly consisting of biting and/or licking of the hindpaw and the tail along with slight hindlimb scratching directed toward the flank. The behavior induced by Ang II (3 pmol) was dose-dependently inhibited by intraperitoneal injection of morphine (0.1-0.3 mg/kg), suggesting that the behavioral response is related to nociception. The nociceptive behavior was also inhibited dose-dependently by i.t. co-administration of losartan (0.3-3 nmol), an Ang II type 1 (AT_1_) receptor antagonist, and SB203580 (0.1-1 nmol), a p38 MAPK inhibitor. However, the Ang II type 2 (AT_2_) receptor antagonist PD123319, the upstream inhibitor of ERK1/2 phosphorylation U0126, and the JNK inhibitor SP600125 had no effect on Ang II-induced nociceptive behavior. Western blot analysis showed that the i.t. injection of Ang II induced phosphorylation of p38 MAPK in the lumbar dorsal spinal cord, which was inhibited by losartan, without affecting ERK1/2 and JNK. Furthermore, we found that AT_1_ receptor expression was relatively high in the lumbar superficial dorsal horn.

**Conclusions:**

Our data show that i.t. administration of Ang II induces nociceptive behavior accompanied by the activation of p38 MAPK signaling mediated through AT_1_ receptors. This observation indicates that Ang II may act as a neurotransmitter and/or neuromodulator in the spinal transmission of nociceptive information.

## Background

Angiotensin II (Ang II), a main bioactive component of the renin-angiotensin system (RAS), plays a critical role in sympathetic regulation, cardiovascular control, fluid balance and hormone secretion (for review, see Refs [1,2]). In the RAS, renin converts angiotensinogen to angiotensin I (Ang I), which in turn is cleaved by angiotensin-converting enzyme (ACE) to Ang II. Ang II mediates its biological effects through Ang II type 1 (AT_1_) receptors and Ang II type 2 (AT_2_) receptors, which are seven transmembrane receptors with approximately 30% amino acid sequence similarity. Most species express a single type of AT_1_ receptors, but two related AT_1A_ and AT_1B_ receptor subtypes are expressed in rodents (for review, see Ref [3]). Ang II is not only generated by circulating ACE, but also produced locally in tissues. The existence of local tissue-based RAS, independent of the classical circulating RAS, has been established in several organs (for review, see Ref [4]). The tissue RAS is characterised by the presence of all RAS components, including angiotensinogen, renin, ACE, Ang I, Ang II and Ang II receptors, and is found in the heart [5], blood vessels [6], kidney [7], pancreas [8], brain [9] and adipose tissue [10]. Evidence indicates that Ang II is involved in the modulation of nociceptive transmission. Namely, Ang II causes hyperalgesia in the caudal ventrolateral medulla (CVLM) [11] and hypoalgesia in the periaqueductal gray (PAG) and the rostral ventromedial medulla (RVM) [12-14]. However, the role of spinal Ang II in the modulation of nociceptive transmission remains unclear.

Ang II acts as an activator of mitogen-activated protein kinase (MAPK) [15-17], a family of Ser/Thr kinases that convert extracellular stimuli into a wide range of cellular responses. The MAPKs include extracellular signal-regulated kinase (ERK) 1/2, c-Jun N-terminus kinase (JNK) and p38 MAPK. These MAPKs have common activation motif (T-X-Y), which are phosphorylated by MAPK kinase. It has been reported that ERK1/2 and JNK are activated in several pain models involving peripheral inflammation, noxious heat and electric stimulation, and that the corresponding nociceptive behaviors are blocked by their respective kinases inhibitor [18-21]. In addition, p38 MAPK, which is activated by cellular stress and proinflammatory cytokines, is considered as a stress-induced kinase and plays a critical role in inflammatory responses. Spinal p38 MAPK is activated by complete Freund's adjuvant (CFA)-induced peripheral inflammation and nociceptive responses accompanying the inflammation are markedly decreased by p38 MAPK inhibitor [22]. Inhibition of p38 MAPK also reduces the mRNA expression of proinflammatory cytokines such as IL-1β, IL-6 and TNFα [22]. These observations indicate that ERK1/2, JNK and p38 MAPK are involved in the facilitation of nociceptive transmission.

We have previously found that intrathecal (i.t.) administration into mice of dynorphin [23,24], spermine [25], D-cycloserine [26] and serotonin releaser [27] produces nociceptive behavior. In the present study, we found that i.t.-administered Ang II also produced nociceptive behavior. To gain insight into the mechanism of Ang II-induced nociceptive behavior, we determined whether Ang II receptor subtypes and MAPK signaling were involved.

## Results

### Behavioral response induced by i.t.-administered Ang II

**Figure 1:**
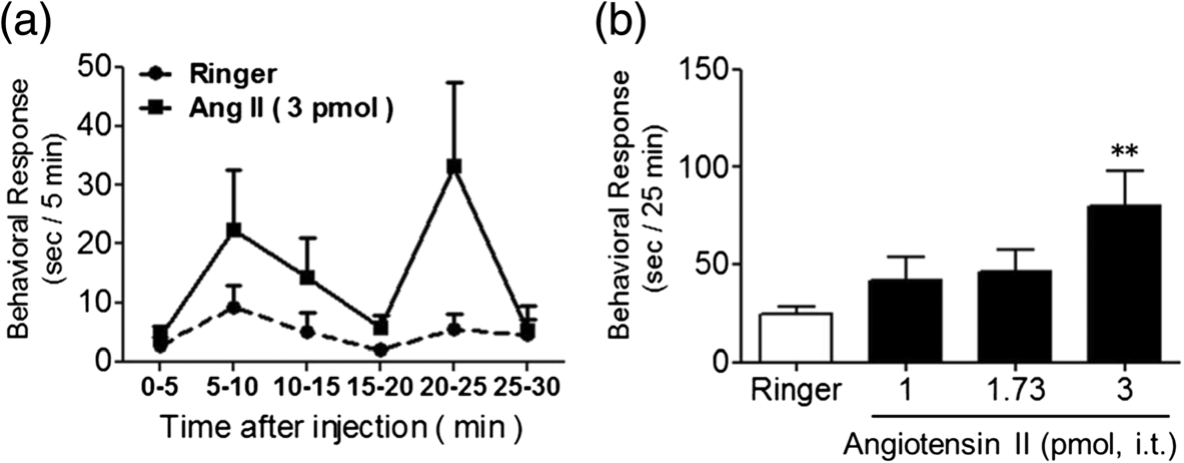
**Scratching, biting and licking responses induced by i.t.-administered Ang II in mice. (a)** Time course of behavioral response induced by Ang II (3 pmol) or Ringer’s solution alone. The ordinate shows the total time of scratching, biting and licking that occurred during each 5 min of measurement. **(b)** Effects of varying doses of Ang II (1–3 pmol/mouse). The duration of scratching, biting and licking induced by Ang II or Ringer’s solution was determined over a 25 min period starting immediately after i.t. injection. Values represent the means ± S.E.M. for groups of 10 mice. *p < 0.05 compared with Ringer controls.

I.t.-administered Ang II (3 pmol) produced a characteristic behavioral response consisting of scratching, biting and licking, which almost disappeared 25 min after the injection (Figure 1a). Two-way repeated-measures ANOVA revealed significant effects of the treatment (*F*_1,18_ = 6.89, *p* < 0.05) and time (*F*_5,90_ = 2.41, *p* < 0.05) but not treatment × time interaction (*F*_5,90_ = 0.89, *p* = 0.17). As seen in Figure 1b, a dose-dependent increase in the total time of scratching, biting and licking for 25 min was observed following i.t. administration of Ang II (1–3 pmol). One-way ANOVA revealed a significant effect of treatment (*F*_3,36_ = 3.47, *p* < 0.05). A post-hoc test demonstrated a significant increase in the behavioral responses induced by injection of Ang II (3 pmol) compared to the Ringer-administered group (*p* < 0.05). Therefore, the latter dose of Ang II was used in subsequent injections which were followed by a 25 min observation period.

**Figure 2:**
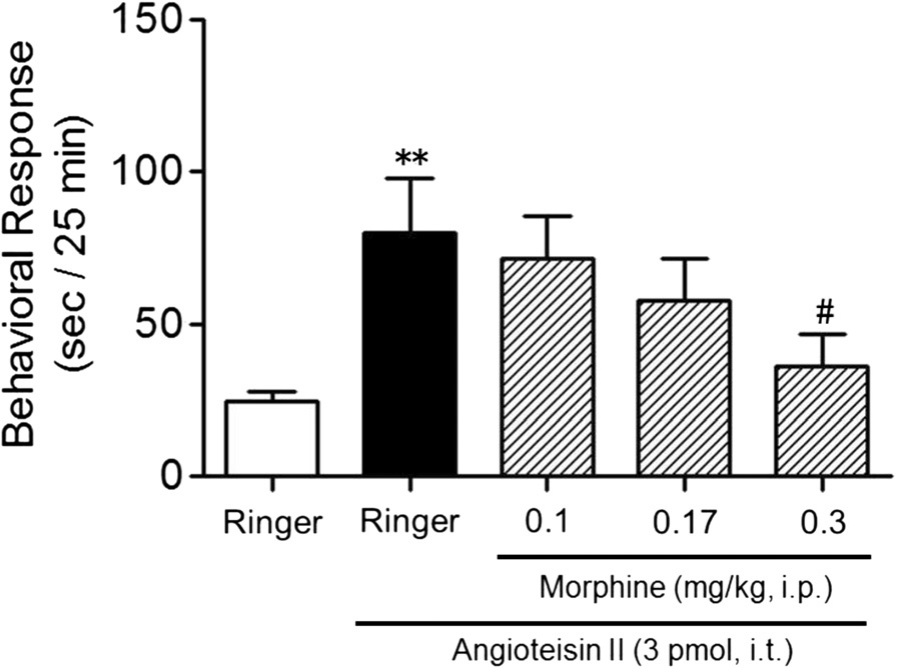
**Effect of morphine on Ang II-induced scratching, biting and licking responses in mice.** Morphine was administered i.p. 5 min prior to injection of Ang II (3 pmol). The duration of scratching, biting and licking induced by Ang II was determined over a 25 min period starting immediately after i.t. injection. Values represent the means ± S.E.M. for groups of 10 mice. **p < 0.01 compared with Ringer controls and # *p* < 0.05 compared with Ang II alone.

To determine whether the Ang II-induced behavior is related to nociception, we examined the effect of a pretreatment with morphine. As shown in Figure 2, morphine (0.1-0.3 mg/kg, i.p.) inhibited the Ang II-induced behavior in a dose-dependent manner with an ID50 value of 0.19 (0.14-0.27) mg/kg, suggesting that the behavioral response is related to nociception (one-way ANOVA analysis, *F*_4,45_ = 3.34, *p* < 0.05; post hoc test, *p* < 0.01 for Ringer versus Ang II, *p* < 0.05 for Ang II versus Ang II plus 0.3 mg/kg morphine).

### Effects of Ang II receptor antagonists on Ang II-induced nociceptive behavior

**Figure 3:**
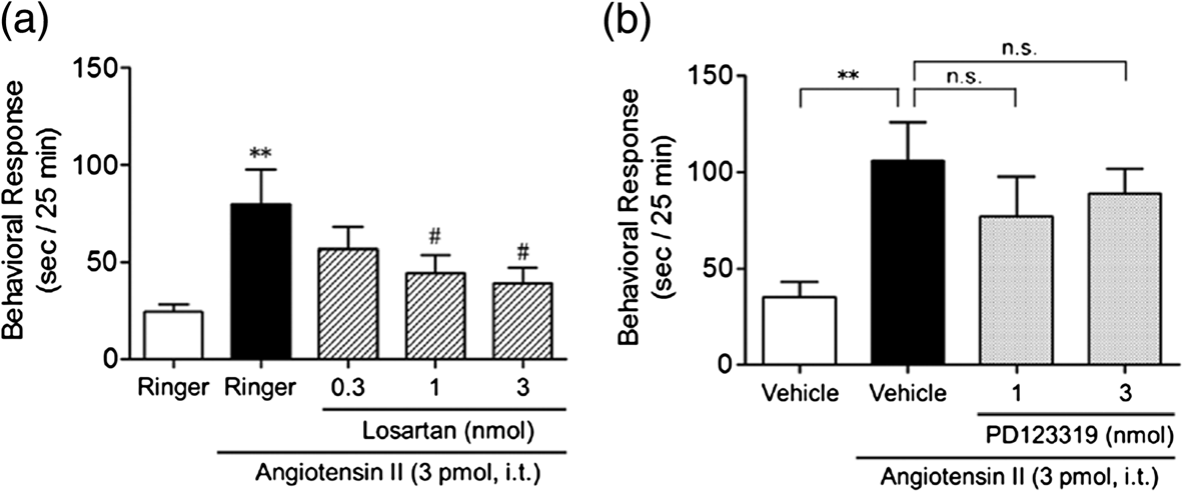
**Effects of Ang II receptor antagonists on i.t. Ang II-induced nociceptive behavior in mice.** Losartan **(a)** or PD123319 **(b)** was co-administered i.t. with Ang II (3 pmol). The duration of scratching, biting and licking induced by Ang II was determined over a 25 min period starting immediately after i.t. injection. Values represent the means ± S.E.M. for groups of 10 mice. **p < 0.01 compared with Ringer or vehicle (6.8% DMSO) controls and # *p* < 0.05 compared with Ang II alone. n.s., not significant.

To determine which type of Ang II receptors is involved in the nociceptive behavior, we compared the effects of losartan, an AT_1_ receptor antagonist, to PD123319, an AT2 receptor antagonist. Losartan (0.3-3 nmol) co-administered i.t. with Ang II caused a dose-dependent inhibition of Ang II-induced nociceptive behavior with an ID50 value of 0.55 (0.47-0.63) nmol (one-way ANOVA analysis, *F*_4,45_ = 3.45, *p* < 0.05; post hoc test, *p* < 0.01 for Ringer versus Ang II, *p* < 0.05 for Ang II versus Ang II plus 1 and 3 nmol losartan, Figure 3a). In contrast, i.t.-administered PD123319 (1 and 3 nmol) did not affect the nociceptive behavior induced by Ang II (one-way ANOVA analysis, *F*_3,36_ = 2.74, *p* < 0.05; post hoc test, *p* > 0.05 for Ang II versus Ang II plus 1 and 3 nmol PD123319, Figure 3b). These results indicate i.t. Ang II-induced nociceptive behavior is mediated through AT_1_ receptors but not through AT_2_ receptors.

### Distribution of AT_1_ receptors in mouse spinal cord

**Figure 4:**
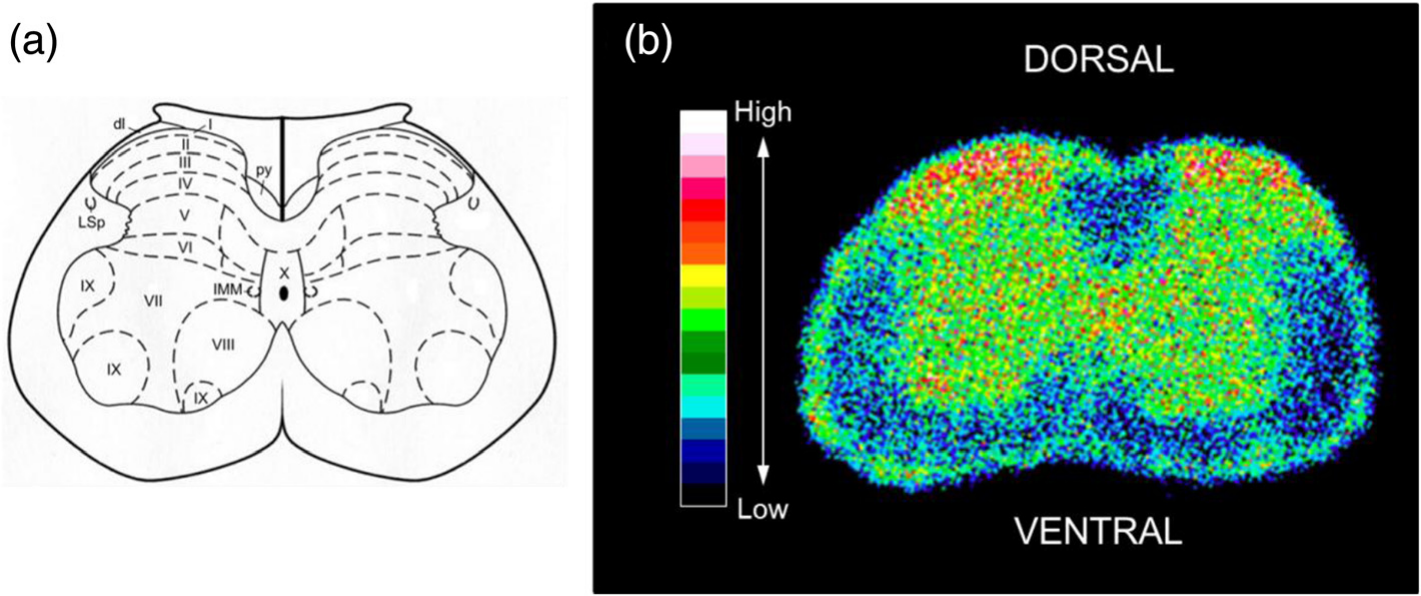
**Distribution of the immunohistochemical fluorescence intensity for AT**_**1 **_**receptors in mouse lumbar spinal cord (L5). (a)** Diagram representing segment L5 of the spinal cord. **(b)** Quantitative immunohistochemical distribution of AT_1_ receptors in the lumbar spinal cord.

The distribution of AT_1_ receptor fluorescence intensity in mouse spinal cord (L5) was determined by microphotometry and categorized into 18 levels (shown as different colors in Figure 4b, with the lowest concentration shown as black and the highest concentration represented by white). Relatively high intensity of AT_1_ receptor fluorescence was seen in the superficial dorsal horn (laminae I and II).

### Effects of MEK and MAPK inhibitors on Ang II-induced nociceptive behavior

**Figure 5:**
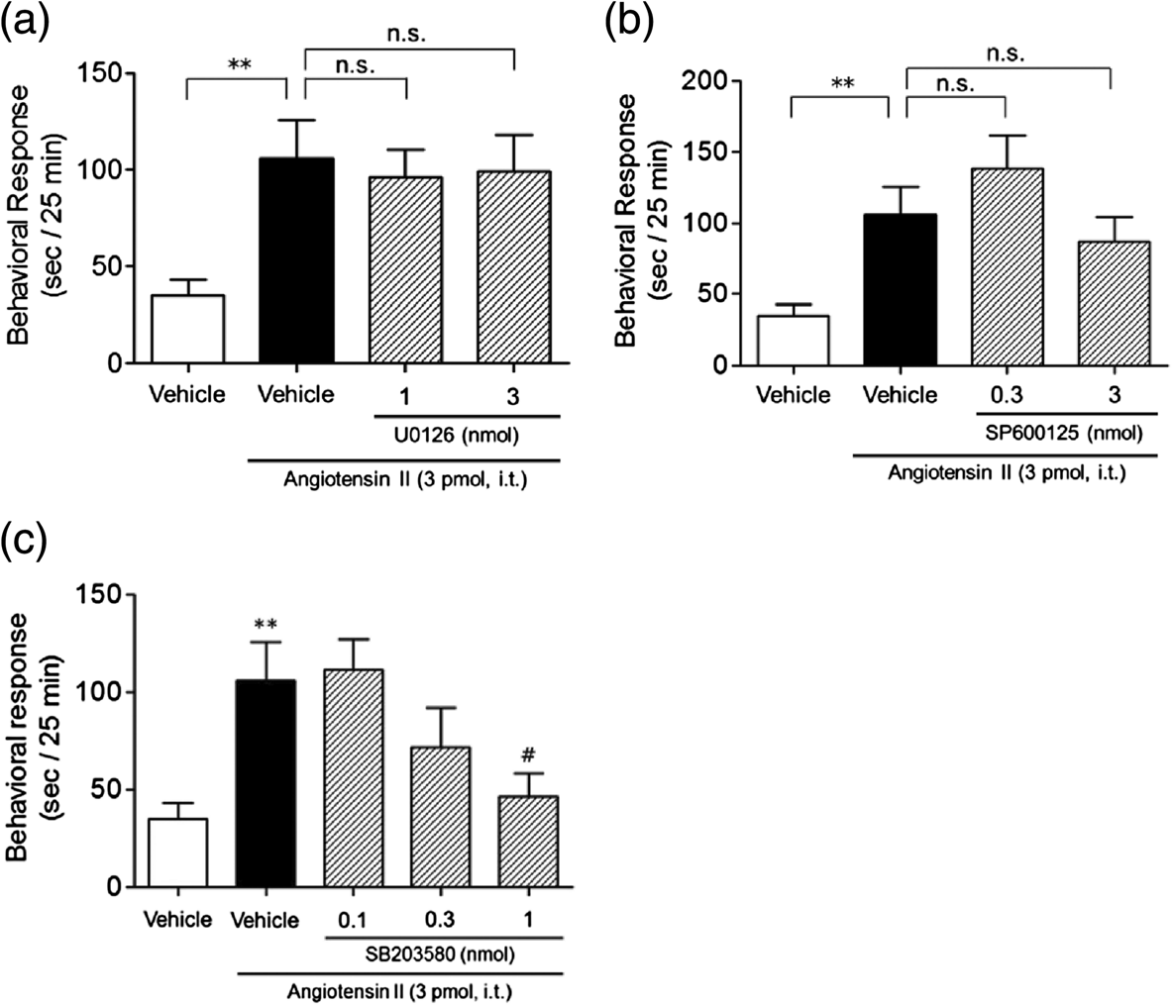
**Effects of MEK and MAPK inhibitors on i.t. Ang II-induced nociceptive behavior in mice.** U0126 **(a)**, SP600125 **(b)** and SB203580 **(c)** were co-administered i.t. with Ang II (3 pmol). The duration of scratching, biting and licking induced by Ang II was determined over a 25 min period starting immediately after i.t. injection. Values represent the means ± S.E.M. for groups of 10 mice. **p < 0.01 compared with vehicle (6.8% DMSO) controls and #*p* < 0.05 compared with Ang II alone. n.s., not significant.

The role of ERK1/2, JNK and p38 MAPK signaling in Ang II-induced nociceptive behavior was examined using the inhibitors U0126, SP600125, and SB203580, respectively. U0126 (1 and 3 nmol) co-administered i.t. with Ang II did not affect the nociceptive behavior induced by Ang II (one-way ANOVA analysis, *F*_3,36_ = 5.11, *p* < 0.01; post hoc test, *p* > 0.05 for Ang II versus Ang II plus 1 and 3 nmol U0126, Figure 5a). Similarly, SP600125 (0.3 and 3 nmol) did not affect the nociceptive behavior induced by Ang II (one-way ANOVA analysis, *F*_3,36_ = 5.82, *p* < 0.01; post hoc test, *p* > 0.05 for Ang II versus Ang II plus 0.3 and 3 nmol SP600125, Figure 5b). On the other hand, i.t.-administered SB203580 (0.1-1 nmol) caused a dose-dependent inhibition of Ang II – induced nociceptive behavior with an ID50 value of 0.34 (0.32-0.37) nmol (one-way ANOVA analysis, *F*_4,45_ = 4.72, *p* < 0.01; post hoc test, *p* < 0.05 for Ang II versus Ang II plus 1 nmol SB203580, Figure 5c). These results suggest that p38 MAPK, but not ERK1/2 and JNK is critically involved in the nociceptive behavior produced by Ang II.

### Phosphorylation of MAPKs in the dorsal spinal cord after i.t. injection of Ang II

**Figure 6:**
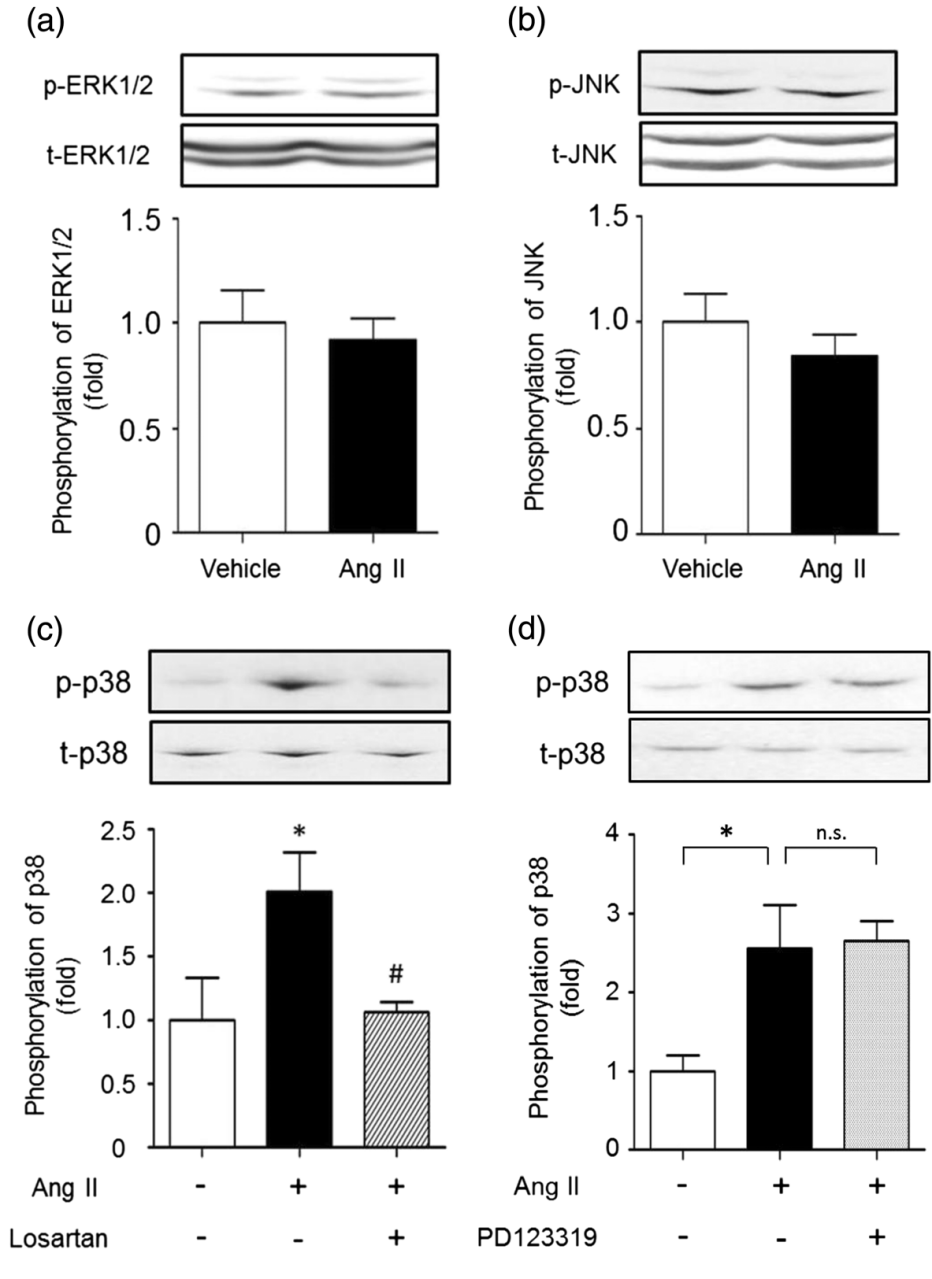
**Alterations in spinal MAPKs phosphorylation by Ang II and the effects of losartan and PD123319.** Dorsal spinal cord samples were taken 10 min after i.t. injection of Ang II (3 pmol). Phosphorylation of ERK1/2 **(a)**, JNK **(b)** and p38 MAPK **(c**, **d)** were examined by Western blotting. Losartan (3 nmol) or PD123319 (3 nmol) was co-administered i.t. with Ang II. Top: representative Western blot of total- and phospho-MAPKs. Bottom: quantification of phospho-MAPKs to total-MAPKs set as 1.0 in the Ringer- or vehicle (6.8% DMSO)-treated group. Each value represents the means ± S.E.M. of 4 mice in each group. *p < 0.05 compared with Ringer or vehicle controls and #*p* < 0.05 compared with Ang II alone. n.s., not significant.

To investigate whether spinal MAPKs were activated by i.t. injection of Ang II (3 pmol), we examined the phosphorylation of ERK1/2, JNK and p38 MAPK in the lumber dorsal cord extracted 10 min after i.t. injection by Western blotting. Ang II did not affect the phosphorylation of ERK1/2 (*t* = 0.47, *p* > 0.05, Figure 6a) and JNK (*t* = 0.97, *p* > 0.05, Figure 6b). As shown in Figure 6c and d, Ang II increased the phosphorylation of p38 MAPK in the lumber dorsal cord. In addition, as seen in Figure 6c, losartan inhibited the p38 MAPK phosphorylation induced by Ang II (one-way ANOVA analysis, *F*_2,9_ = 4.50, *p* < 0.05; post hoc test, *p* < 0.05 for Ang II versus Ang II plus 3 nmol losartan). In contrast, PD123319 did not affect the p38 MAPK phosphorylation induced by Ang II (one-way ANOVA analysis, *F*_2,9_ = 6.99, *p* < 0.05; post hoc test, *p* > 0.05 for Ang II versus Ang II plus 3 nmol PD123319, Figure 6d). These results indicate that i.t.-administered Ang II produces p38 MAPK phosphorylation mediated through AT_1_ receptors but not through AT_2_ receptors in the lumber dorsal cord.

## Discussion

In the present study, we demonstrated for the first time that i.t.-administered Ang II in mice induced a characteristic behavioral response mainly consisting of biting and/or licking of the hindpaw and the tail along with slight hindlimb scratching directed toward the flank, indicative of nociceptive responses, accompanied by the activation of p38 MAPK mediated through AT_1_ receptors.

Ang II was originally discovered as a potent vasoconstrictor, while recent studies have shown that Ang II affects a wide range of central and peripheral components of sensory systems [13,28,29]. It has been demonstrated that the administration of Ang II either i.c.v. or directly in key components of the supraspinal pain modulatory system, namely the PAG or RVM (for review, see Ref [30]), induces antinociceptive effects, which are reversed by losartan [12,13,31,32]. On the other hand, Marques-Lopes et al. [11] have recently reported that the microinjection of Ang II into the CVLM induces hyperalgesia through AT_1_ receptors, and that the effect of Ang II on spinal nociceptive processing is likely indirect, since few AT_1_ receptor-expressing CVLM neurons were found to project to the spinal cord. These reports lead us to suggest that supraspinal Ang II may participate in both inhibition and facilitation of the nociceptive transmission and its effect is region-dependent. However, the role of Ang II in the modulation of nociceptive transmission in the spinal cord has not been reported until this study. Therefore, it is important to clarify the role of spinal Ang II in the modulation of nociception.

Recently, it has been reported that Ang II is colocalized with calcitonin gene-related peptide (CGRP) and substance P, the neuropeptides established as the key regulators of sensory transmission and nociception, in rat and human dorsal root ganglia (DRG) [33]. Takai et al. [34] have revealed that repeated oral administration of AT1 receptor antagonist and ACE inhibitors show antinociceptive effect in hot-plate test. Furthermore, we have found that i.t.-administered losartan produces antinociceptive effect in a mouse formalin test (data not shown). These findings suggest that Ang II may act as a neurotransmitter and/or neuromodulator in the transmission of nociceptive information in the spinal cord. In the present study, we found that i.t.-administered Ang II (3 pmol) produced a nociceptive behavior consisting of scratching, biting and licking. We also observed that the Ang II-induced nociceptive behavior was inhibited by losartan but not by PD123319, indicationg that receptor type 1 and not type 2 for Ang II is involved. Regarding the distribution of spinal AT_1_ receptors, Pavel et al. [35] have reported that the receptors are present in high density in the lumbar superficial dorsal horn (laminae I and II) using autoradiography in rat. In this study, we also detected a relatively high intensity of fluorescence for AT_1_ receptors in the mouse lumbar superficial dorsal horn. Our results obtained with behavioral and immunohistchemical experiments suggest that spinal AT_1_ receptors are responsible for the nociceptive response.

Ang II induced two peaks of nociceptive behavior, one at 5–10 and the other 20–25 min after injection, although there was no significant difference between time × treatment interaction. The hydrolysis of Ang II by a homogenate of rat ventrolateral PAG forms Ang III, a major hydrolysis product, Ang IV, Ang (1–7) and Ang (1–4) [36]. Moreover, microinjection of Ang III into the ventrolateral PAG produces the antinociceptive effect mediated through AT_1_ and AT_2_ receptors [36]. Therefore, we may speculate that in our time course experiment, Ang II is responsible for the first peak while Ang III generated from Ang II is responsible for the second peak.

ERK1/2, JNK and p38 MAPK are phosphorylated in the presence of Ang II in mouse atrial fibroblasts [15] and natural killer cells [16], while only ERK1/2 and p38 MAPK but not JNK are phosphorylated by Ang II in RVM [17]. In addition, Sung et al. [37] have reported that i.t.-administered IL-1β activates only p38 MAPK without affecting ERK1/2 and JNK in the spinal cord. Similarly, in this study, only the spinal p38 MAPK was activated after i.t. administration of Ang II, although the ERK1/2, JNK and p38 MAPK were constitutively expressed in the spinal cord. There are four p38 MAPK isoforms: p38α, p38β, p38γ and p38δ. Whereas p38α and p38β are two of the major isoforms in the mature nervous system, p38α is the most abundant isoform in DRG neuron and spinal cord (for review, see [38]). Therefore, we used SB203580 to inhibit p38 MAPK signaling in the spinal cord since it can inhibit the activity of both p38α and p38β isoforms [39]. In this study, the behavioral observation revealed that Ang II-induced nociceptive response was almost completely inhibited by SB203580. On the other hand, neither U0126 nor SP600125 affected the Ang II-induced nociceptive behavior. Ample evidence suggest that the spinal p38 MAPK is involved in several types of pain. Phosphorylation of spinal p38 MAPK has been observed not only in neuropathic pain models such as chronic constriction injury [40,41] and spinal nerve ligation [42-44], but also in peripheral inflammation induced by CFA [22], bee-venom [45], formalin [46-48] and capsaicin [48]. Moreover, i.t. administration of *N*-methyl-D-aspartate (NMDA) produces thermal hyperalgesia through spinal p38 MAPK phosphorylation [49]. Taken together with these previous reports, our present results indicate that the phosphorylation of spinal p38 MAPK, but not of the other MAPKs, is involved in Ang II-induced nociceptive behavior. In addition, since the nociceptive behavior arises rapidly and declines within 25 min to resemble controls, we suggest that the phosphorylation of p38 MAPK leads to the behavior via non-transcriptional mechanisms. Mizushima et al. [50] have reported that intraplantar injection into rats of capsaicin induces phosphorylation of p38 MAPK in DRG neurons and thermal hyperalgesia which peak at 2–5 min after injection. Although the specific target proteins of p38 MAPK are not clearly identified, p38 MAPK signaling pathway leads to Ang II-induced nociceptive behavior through post-transcriptional modifications of kinases, receptors and ion-channels.

Finally, we examined the effects of Ang II receptor antagonists on p38 MAPK phosphorylation in the dorsal spinal cord. Whereas p38 MAPK phosphorylation was inhibited by losartan, it was resistant against PD123319, and these results were consistent with those of the behavioral experiments. It has been reported that Ang II increases the phosphorylation of p38 MAPK in cultured rat neonatal cardiomyocytes, which is attenuated by losartan similarly to SB205380, a p38 MAPK inhibitor, and p38 siRNA [51]. Taken together, the present results suggest that phosphorylation of p38 MAPK mediated through AT_1_ but not AT_2_ receptors contributes to i.t. Ang II-induced nociceptive behavior.

## Conclusions

In conclusion, our data show that i.t.-administered Ang II induces nociceptive behavior accompanied by p38 MAPK phosphorylation mediated through spinal AT_1_ receptors. Moreover, it is suggested that Ang II may be a neurotransmitter and/or neuromodulator in the transmission of nociceptive information in the spinal cord.

## Methods

### Animals

Male ddY strain mice (weighing 22–24 g, Japan SLC, Japan) were used in all experiments. Mice were housed in cages with free access to food and water under conditions of constant temperature (22 ± 2°C) and humidity (55 ± 5%), on a 12 h light–dark cycle (lights on: 08:00 to 20:00). Groups of 10 mice for behavioral experiments and 4 mice for Western blotting and immunohistchemical experiments were used in single experiments. All experiments were performed following the approval from the Ethics Committee of Animal Experiment in Tohoku Pharmaceutical University and according to the National Institutes of Health Guide for the Care and Use of Laboratory Animals. Efforts were made to minimize suffering and to reduce the number of animals used.

### Intrathecal injections

The i.t. injections were made in unanaesthetized mice at the L5, L6 intervertebral space as described by Hyden and Wilcox [52]. Briefly, a volume of 5 μl was administered i.t. with a 28-gauge needle connected to a 50-μl Hamilton microsyringe, the animal being lightly restrained to maintain the position of the needle. Puncture of the dura was indicated behaviorally by a slight flick of the tail.

### Behavioral observation

Approximately 60 min before the i.t. injection, the mice were habituated to an individual cage (22.0 × 15.0 × 12.5 cm) which was also used as the observation chamber after injection. Immediately after the i.t. injection, the mice were placed in the transparent cage and the accumulated response time of hindlimb scratching directed toward the flank, biting and/or licking of the hindpaw and the tail was measured for 25 min with the exception of the 30 min time course experiment in which the response was divided into 5 min intervals.

### Drugs and antibodies

The following drugs and chemicals were used: Ang II (Peptide Institute, Japan); morphine hydrochloride (Sankyo, Japan); losartan potassium (LKT Laboratories, USA); 1-[[4-(dimethylamino)-3-methylphenyl]methyl]-5-(diphenylacetyl)-4,5,6,7-tetrahydro-1*H*-imidazo[4,5-*c*]pyridine-6-carboxylic acid ditrifluoroacetate (PD123319), 1,4-diamino-2,3-dicyano-1,4-*bis*[2-aminophenylthio]butadiene (U0126), 4-[5-(4-fluorophenyl)-2-[4-(methylsulphonyl) phenyl]-1*H*-imidazol-4-yl]pyridine hydrochloride (SB203580 hydrochloride) (Tocris Biosciense, USA); anthra (1,9-*cd*) pyrazol-6(2*H*)-one, 1,9-pyrazoloanthrone (SP600125) (Alexis, USA); sodium pentobarbital (Dainippon Sumitomo Pharma, Japan); antibodies against ERK1/2, phospho-ERK1/2, JNK, phospho-JNK, p38 MAPK, phospho-p38 MAPK, and horseradish peroxidase (HRP)-conjugated goat anti-rabbit IgG antibody (Cell Signaling Technology, USA); anti-AT_1_ receptor antibody (Alpha Diagnostic, USA); enhanced chemiluminescence (ECL) assay kit (GE Healthcare, England). For i.t. injections, Ang II and losartan were dissolved in Ringer’s solution. PD123319, U0126, SB203580 and SP600125 were dissolved in Ringer’s solution containing 6.8% dimethyl sulfoxide (DMSO). When the effects of Ang II receptor antagonists and MAPK-related inhibitors were tested, they were co-injected with Ang II in a volume of 5 μl. Morphine was dissolved in physiological saline and administered intraperitoneally (i.p.) 5 min prior to injection of Ang II.

### Immunohistochemical staining

Spinal cords for measurement of AT_1_ receptors were prepared within 24 h following delivery. Mice were anesthetized with sodium pentobarbital (50 mg/kg, i.p.) and perfused through the heart with ice-cold phosphate-buffered saline (PBS, pH 7.2), immediately followed by a fixative containing 4% paraformaldehyde (Sigma–Aldrich, USA) and 0.2% glutaraldehyde (Nacalai Tesque, Japan) in PBS. Spinal cords (lumbar 5; L5) were then postfixed with the same fixative solution at 4°C for 1 h and then placed in a 20% sucrose-buffered solution at 4°C for 12 h. Tissues were frozen on dry ice and cut into 20 μm-thick coronal sections on a cryostat (Micro-edge Instruments Co. Ltd., Germany). The immunohistochemical staining procedure was carried out as previously described [53]. Briefly, a rabbit anti-AT_1_ receptor antibody (diluted 1:100 with PBS and 5% normal goat serum (NGS); Millipore Co., USA) was applied to spinal cord slices, which were then incubated at 4°C for 12 h. The secondary antibody consisted of FITC-labeled anti-rabbit IgG goat serum (diluted 1:200 with PBS; Millipore Co.), and was allowed to react in the dark at room temperature for 2 h. The stained sections were mounted in Dako Fluorescence Mounting Medium (Dako North America, USA), and kept at 4°C in a dark room until measurements were carried out. The distribution of AT_1_ receptor immunofluorescence intensities was quantitatively analyzed using a MapAnalyzer (Yamato Scientific Co., Japan). The background value, including non-specific fluorescence originating from glutaraldehyde, was subtracted photometrically from the total fluorescence intensity value at each point measured.

### SDS-polyacrylamide gel electrophoresis and immunoblotting

Samples used for immunoblotting were prepared as follows. At 10 min after i.t. injection, mice were decapitated and the whole spinal cord was taken by pressure expulsion with physiological saline. The dorsal part of lumbar spinal cord was dissected quickly on ice-cooled glass dish. The tissue samples were homoginaized in 0.15 ml of CelLytic™ MT Manmalian Tissue Lysis/Extraction Reagent (Sigma Aldrich, USA) and centrifuged the lysis sample at 15,000× *g* for 15 min at 4°C. Supernatants were dissolved in 4 × Laemmli sample buffer (300 mM Tris–HCl pH 6.8, 8% SDS, 40% glycerol, 12% 2-mercaptoethanol and 0.012% bromophenol blue), and boiled at 95°C for 10 min. Electrophoresis was performed on 10% acrylamide gels. Proteins were transferred electrically from the gel onto a polyvinylidene difluoride membrane (Bio-Rad Laboratories, Japan) by the semi-dry blotting method. The blots were blocked for 30 min with 5% skim-milk in Tris-buffered saline supplemented with 0.1% Tween-20, and incubated with primary antibodies overnight at 4°C. The blots were washed several times and then incubated at room temperature for 2 h with a secondary antibody (HRP-conjugated anti-rabbit IgG antibody). Blots were developed using an enhanced chemiluminescence assay kit, and visualized by chemiluminescence on Hyper-film ECL. The densities of the bands were analyzed by densitometry (Image-J 1.43u, National Institute of Health).

### Statistical methods

Data were expressed as mean ± SEM. The ID_50_ values with 95% confidence limits were calculated for reduction in Ang II-induced scratching, biting and licking response by a computer-associated curve-fitting program (GraphPad Prism; GraphPad Software, USA). The significant differences were analyzed by a one-way or two-way analysis of variance (ANOVA), followed by Fisher’s PLSD test for multiple-comparisons. Student’s *t* test was used for comparisons between two groups. In all comparisons, *P* < 0.05 was considered statistical significance.

## Abbreviations

ACE: angiotensin-converting enzyme; Ang: Angiotensin; AT1: Ang II type 1; AT2: Ang II type 2; CFA: complete Freund's adjuvant; CGRP: calcitonin gene-related peptide; CVLM: caudal ventrolateral medulla; DMSO: dimethyl sulfoxide; DRG: dorsal root ganglia; ERK: extracellular signal-regulated kinase; ID50: inhibitory dose 50%; i.p.: intraperitoneal; i.t.: intrathecal; JNK: c-Jun N-terminus kinase; MAPK: mitogen-activated protein kinase; NMDA: *N*-methyl-D-aspartate; PAG: periaqueductal gray; RAS: renin-angiotensin system; RVM: rostral ventromedial medulla.

## Competing interests

The authors declare that they have no competing interests.

## Authors’ contributions

WN designed, performed the experiments and wrote the manuscript. ON performed immunohistchemical experiment and analyzed the data. FY, SK and SY contributed to design of experimentation. MI and TT supervised the experiments. KT supervised the experiments, and participated in their design and coordination, and helped to draft the manuscript. All authors read and approved the final manuscript.
